# The Influence of Azidated Glycerol as a New Low Temperature Stabilizer on the Colloidal Properties of Natural Rubber Latex: For Latex Marine Transportation

**DOI:** 10.3390/polym15030698

**Published:** 2023-01-30

**Authors:** A. S. Siti Nuraya, A. A. Azniwati, A. Baharin, A. R. Azura, M. F. Yhaya

**Affiliations:** 1 School of Industrial Technology, Universiti Sains Malaysia, Minden 11800, Penang, Malaysia; 2 Green Biopolymer, Coatings & Packaging Cluster, School of Industrial Technology, Universiti Sains Malaysia, Minden 11800, Penang, Malaysia; 3 School of Materials and Mineral Resources Engineering, Universiti Sains Malaysia, Nibong Tebal 14300, Penang, Malaysia; 4 Biomaterials and 3D Visualisation (BioM3D) Laboratory, School of Dental Sciences, Health Campus, Universiti Sains Malaysia, Kubang Kerian 16150, Kelantan, Malaysia

**Keywords:** azidated glycerol, low temperature stabilizer, natural rubber latex, colloid stability, freeze–thaw

## Abstract

Natural rubber latex (NRL) is normally transported to a destination in colloid/liquid form. It requires large storage containers such as drums and the probability of latex leakage during transportation is high. This is prevalent especially when transporting latex by sea. To prevent latex spillage, the liquid form of NRL is transformed into solid/frozen latex by freezing. However, the coagulation/destabilization of NRL by freezing has been acknowledged as a problem for years. Therefore, this study proposed a new low temperature stabilizer named azidated glycerol (AG) to be incorporated in NRL liquid before the freezing process. AG was prepared by a chemical reaction of pure glycerol with sodium azide. NRL containing AG was then frozen at a temperature of −4 °C. After 24 h of freezing, the frozen latex was thawed at ambient temperature for 1 h followed by heating in a water bath at 40 °C for another 1 h. The regenerated latex was then allowed to stand at room temperature before testing. The effect of AG on the colloid properties before and after the freeze–thaw processes was studied. The production of AG was confirmed by the appearance of a peak in the range of 2160–2120 cm^−1^, corresponding to N=N=N stretching, confirming the introduction of an azide group into the glycerol molecule. Modifying NRL with AG did not significantly influence the TSC of latex. Increasing the AG content up to 0.4 phr resulted in an increase in MST from 699 s to 828 s. An AG content of 0.2 phr resulted in the highest anionically stabilized latex as indicated by zeta potential values of −59.63 mV (before freezing) and −56.27 mV (after thawing). It is concluded that the AG produced in this study can be used as an anti-freeze stabilizer for NRL and is suitable for latex marine transportation.

## 1. Introduction

In latex technology, the addition of stabilizer is mainly for the stabilization of rubber particles. As well known, fresh latex contains a protective layer of proteins and lipids which act as a natural stabilizer [1]. However, this layer is partially removed during the processing of NRL before goods production. Thus, additional stabilizers are added in order to enhance the colloid stability of NRL during processing. In general, anionic type stabilizers mainly confer mechanical stability, meanwhile the use of non-ionics is more extensive as they can be used to confer both mechanical and chemical stability to the rubber particles [2]. Anionic stabilizers of interest in latex technology are conveniently sub-classified as carboxylates, sulphonates, and sulphates, according to the nature of the ionized site. Oleates which are sub-classified under carboxylate stabilizers have been extensively used in latex emulsions due to their outstanding ability to maintain the colloid behaviour of lattices [3].

The use of potassium oleate (PO) as a typical stabilizer in NRL dipping products is a common practice to maintain the colloidal properties of the NRL compound. However, higher loading of PO in latex formulations to retain colloidal properties after the freezing and thawing processes is associated with debilitating skin conditions such as irritation and itching. This is because PO is a soap-based stabilizer, that is mainly used in detergents and can cause contact allergies when exposed to the skin for long periods of time [4,5]. By replacing soap-based stabilizers with new stabilizers such as those generated in this study, skin allergies could be prevented. 

The typical stabilizers used in NRL can only enhance the colloid stability of NRL during processing and transportation/exportation to temperate countries. However, these stabilizers cannot prevent the particles from coalescing at low temperatures [6]. This is because NRL is very sensitive to freezing temperatures and there is no protection from frost [3]. Prevention of particle coalescence after the freezing and thawing processes is important not only to export latex to temperate countries, where the temperature could drop below zero during winter, but because freezing could be used as a method of storing the latex. Storage of latex by freezing could prevent bacteria from attacking the latex, and thus the latex could be free from preservatives. In this study, the new low temperature stabilizer, azidated glycerol (AG), was added in NRL liquid before formation of solid rubber (frozen latex) for storage and transportation purposes. Thus, the storage and transportation were carried out in solid form of NRL. 

**Figure 1:**
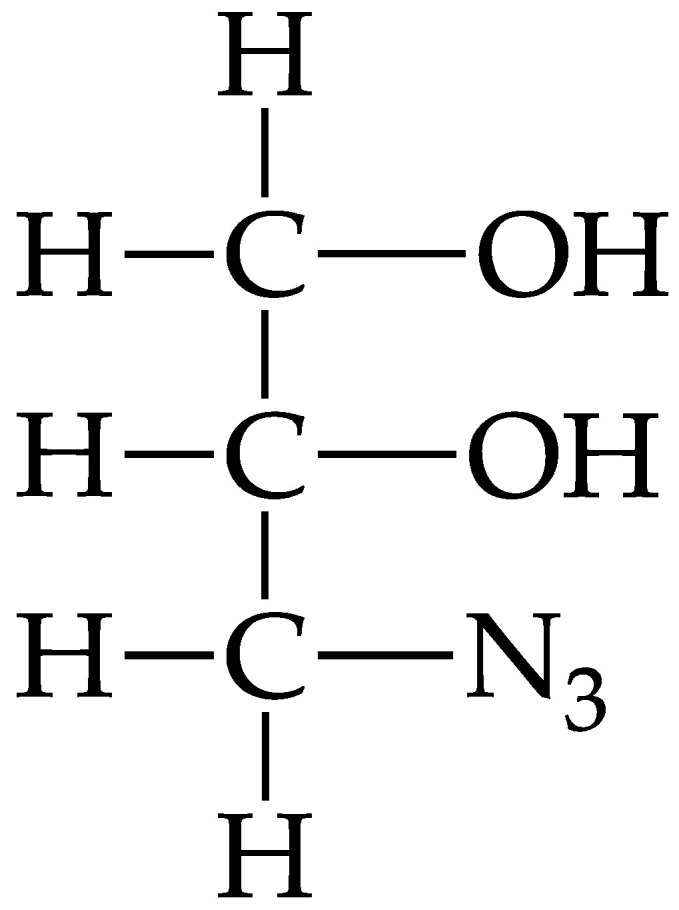
Chemical structure of azidated glycerol (AG).

In recent decades, there have been no active studies performed on the freezing stability of NRL, and the latest study on freezing NRL was in 1969 by Cockbain and co-workers [7]. As a comparison, a few papers on the freezing or low temperature properties of synthetic rubber, however, have been published [8,9,10]. To expand the knowledge on NRL freezing properties, in this study, AG was generated by the modification of pure glycerol with an azide group from sodium azide. Glycerol is a simple polyol compound that is colorless, odorless, viscous, and non-toxic liquid. Owing to the presence of three hydroxyl groups (OH), glycerol is miscible with water and is hygroscopic [11]. Modification of glycerol with an azide group is done by replacing one of the hydroxyl (OH) groups with an azide group to transform the glycerol into AG to make it more compatible with NRL. The chemical structure of AG is shown in Figure 1.

The primary objective of this study is to produce frozen NRL (ice cream-like texture) that can be thawed and re-frozen again for the subsequent manufacturing processes. Frozen NRL can be stored and transported to any destination without worrying about the storage stability of the latex.

## 2. Materials and Methods

High ammonia (HA) (with 61% total solid content (TSC)), natural rubber latex (NRL), curative agents (sulphur, zinc oxide (ZnO), and zinc diethyldithiocarbamate (ZDEC)), antioxidant 2,2-Methylene-bis-(4-methyl-6-tert-butyl phenol), and calcium nitrate (CaNO_3_) used in this study were purchased from ZARM Scientific and Supplies (Malaysia) Sdn. Bhd. 

Meanwhile, the stabilizer used in this study, AG, was produced in the Latex Laboratory, Universiti Sains Malaysia (USM) Engineering Campus, Malaysia. 

### 2.1. Preparation of Azidated Glycerol 

The AG was prepared by a two-step of chemical reaction comprising tosylation and azidation processes, as described below. The chemical reactions were conducted in a fumehood as a safety precaution.

#### 2.1.1. Tosylation of Glycerol 

Tosyl chloride (10 g) was added to a 50 cm^3^ round-bottomed flask containing 4.839 g of glycerol while stirring with a magnetic bar. Triethylamine (TEA) (6.06 g) was added into the mixture as a base. After 24 h of stirring at room temperature, the reaction mixture was stopped, washed with ethanol, and filtered by using a chromatography column containing silica gel as a stationary phase for adsorbing substances. The product was then evaporated to remove excess ethanol by a rotary evaporator.

#### 2.1.2. Azidation of Tosyl Glycerol 

Tosylated glycerol (14 g) and sodium azide (3.699 g) were added to 10 cm^3^ distilled water as a solvent in a 50 cm^3^ round-bottomed flask while stirring with a magnetic bar. The reaction was then heated and maintained at 70 °C for 48 h with stirring. 

After that, the reaction was stopped and cooled to room temperature, then it was washed with ethanol and filtered by using a chromatography column containing silica gel as a stationary phase for adsorbing substances. The excess water and ethanol were evaporated by using a rotary evaporator at 90 °C until no more liquid was collected. The resulting highly viscous liquid is the AG. It was added to NRL after storing overnight at room temperature to make sure all the chemical reactions had stopped and were stable.

### 2.2. Preparation of Natural Rubber Latex with Variable Loading of Azidated Glycerol

The received HA NRL had various TSCs. Thus, HA NRL concentrates were diluted to 50% TSC before mixing with a variable loading of AG. The dilutions were maintained at 50% as an early experiment found that at a higher percent of TSC, the latex gelled after thawing. Furthermore, direct use of the latex in its current form, for example, in electron beam vulcanization of latex, can be explored in future studies [12]. After that, NRL with a variable loading of AG was prepared, based on the formulations shown in Table 1, by mixing the AG with NRL at room temperature while continuously stirring at 250 rpm for 2 h. NRL without AG content (0 phr) was also prepared as a control sample. 

The resulting NRL mixtures were allowed to stand at room temperature for 24 h in airtight containers to allow any trapped air bubbles to escape. Then, the mixtures were poured into smaller airtight plastic containers at the same amount for each loading of AG (~300 g). After that, the containers were placed in a freezer at a temperature of −4 °C for 24 h. Mixtures without freezing were also prepared as a comparison/control condition.

### 2.3. Thawing of Frozen Latex

The frozen latex mixtures were thawed by allowing them to stand at room temperature for 1 h followed by heating in a water bath at 40 °C for another 1 h. The resulting thawed latex or regenerated latex was then allowed to stand at room temperature for 24 h before further tests were carried out. This thawing condition was used as early experiments showed that slow thawing carried out at room temperature or accelerated thawing at 70 °C both resulted in latex coagulation.

### 2.4. Evaluation of Colloidal Properties 

The colloidal properties of the NRL colloid before and after the freeze–thaw processes, such as the total solid content (TSC), pH value, viscosity, and mechanical stability time (MST), were evaluated.

#### 2.4.1. Total Solid Content (TSC) 

About 2.0 ± 0.5 g of the latex mixtures was poured into a Petri dish. The contents of the dish were gently swirled to ensure the latex covered the dish’s bottom. After that, the dish containing latex was heated in an oven at 105 ± 5 °C until the sample lost its whiteness (around 2 h). The dish containing latex was cooled at ambient temperature before weighing. The drying procedure was repeated until a constant weight was achieved. The TSC was calculated using the formula below:

TSC = [m_1_/m_o_] × 100% (1)

where m_o_ is the mass in grams of the test portion and m_1_ is the mass in grams of the dried material.

#### 2.4.2. pH 

The pH value of latex was determined using a pH meter, SI Analytic 285204060 Lab 850 series, that was equipped with an electrode (BlueLine 56 pH Electrode). The pH meter was calibrated with standard buffer solutions at room temperature according to the manufacturer’s instruction manual. The pH of the thawed HA NRL was measured by placing a glass electrode into a 250 cm^3^ beaker containing 50 g of latex at room temperature. The pH values of the latex mixtures before freezing were also measured.

#### 2.4.3. Viscosity 

The viscosity of the latex mixtures before and after the thawing process were measured by a Brookfield viscometer (02072 Model LVF and LVT). About ~200 g of the latex sample was poured into a 250 cm^3^ beaker. Spindle number 2 was selected and attached to the viscometer shaft, and the guard was securely attached to the motor of the viscometer. The spindle was placed at the centre of the beaker and the viscosity was measured at a speed of 60 rpm at room temperature.

#### 2.4.4. Mechanical Stability Time (MST)

The MST of latex samples was measured by using a natural rubber latices mechanical stability apparatus (TO B.S. 1672: 1972). About ~80 g of the latex sample was weighed into the test container. The spindle of the MST apparatus was lowered until the top layer of the latex reached the mark on the spindle. The MST was measured at a speed of 14,000 ± 200 rpm at room temperature. The MST is the time taken for the formation of flocculates in the latex sample. The time was measured by using a stopwatch which was started simultaneously with the MST apparatus. The first appearance of flocculates was taken as the endpoint. After the endpoint was reached, the latex was stirred for another 15 s to observe the increase in the amount of flocculates to confirm the endpoint.

#### 2.4.5. Transmission Electron Microscopy (TEM)

The latex was diluted to 0.05% total solid content by adding 4.4 cm^3^ of distilled water to 0.6 g of latex to produce a total volume of 5 cm^3^, and a drop of 2% osmium tetroxide (OsO_4_) was added and the mixture was stirred for 24 h. One drop of the stained latex was deposited onto the glow-discharged carbon-coated grids and allowed to dry for about 3 min. After that, the grids were scanned with a Philips CM 12 CRYO transmission electron microscope. 

#### 2.4.6. Particle Size Distribution 

The particle size distributions of the HA NRL and the regenerated HA NRL were measured using a Malvern Zetasizer Ver. 7.11 at the Institute for Research in Molecular Medicine (INFORM), Universiti Sains Malaysia. From the output of the machine, the mean particle diameter (d50) was taken as the average particle size.

#### 2.4.7. Fourier Transform Infrared (FTIR) Analysis 

Fourier transform infrared (FTIR) spectrometry (Perkin Elmer, Model Spectrum One), manufactured by Perkin-Elmer Corporation, New York Pacific was used to characterize and identify the inorganic and organic molecules. The analysis was performed at room temperature within the typical wave number range of 4000 cm^−1^ to 500 cm^−1^ in transmission mode. 

#### 2.4.8. Zeta Potential 

Zeta potential is a measure of the magnitude of the electrostatic or charge repulsion/attraction between particles and is one of the fundamental parameters known to affect stability. The zeta potential value was measured using a Malvern Zetasizer Ver. 7.11 at the Institute for Research in Molecular Medicine (INFORM), Universiti Sains Malaysia. The Smoluchowski model was used to convert electrophoretic mobility to zeta potential as particles were expected to be larger than 200 μm and the latex was in polar media (water).

## 3. Results

### 3.1. Properties of Azidated Glycerol 

**Figure 2:**
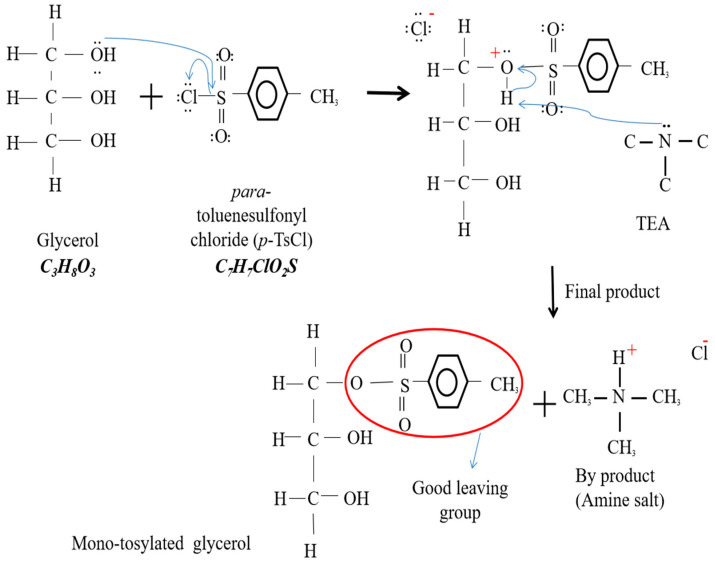
Tosylation reaction of tosylated glycerol production.

To produce AG, a chemical reaction of two steps was involved; namely, tosylation and azidation reactions. Glycerol is a polyhydric alcohol, and by having three free hydroxyls (OH) groups, it can undergo many chemical reactions, and some compounds can be used to produce other derivatives. It has been found that the alcohol hydroxyl group (poor leaving group) can be converted into a good leaving group by replacing the hydroxyl group with a tosyl group [13,14,15]. Para-toluenesulphonyl chloride (p-TsCl) has been widely used as a tosylating agent over tosyl anhydride and p-tolunenesulphonic acid [16]. p-TsCl contains sulphur which is bound to two oxygens and a chlorine. Oxygen and chlorine are more electronegative than sulphur, so sulphur has a partial positive charge. Meanwhile, the oxygen in the hydroxyl group of glycerol has a partial negative charge, so it is attracted to the sulphur atom. Therefore, the oxygen atom will attack the sulphur atom, and the chlorine atoms are removed from the p-TsCl. However, hydrogen atoms are still attached to oxygen and usually, triethylamine (TEA) is used as a strong nucleophilic base. TEA will accept hydrogen atoms and form an amine salt (by-product). Additionally, at the end of the reaction, the final product produced is called tosylated glycerol. The mechanism of the tosylation reaction is illustrated in Figure 2.

**Figure 3:**
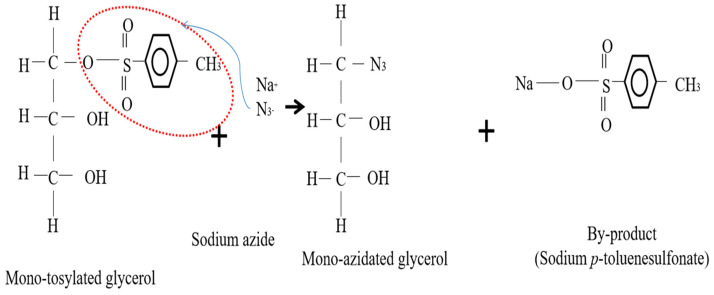
Azidation reaction for the production of azidated glycerol (AG).

After then, the tosylated glycerol was reacted with sodium azide (NaN_3_); this chemical reaction is called an azidation process. The azidation mechanism to produce AG from tosylated glycerol is shown in Figure 3. This reaction involved the replacement of the tosylate leaving group with the azide ion (N^3−^) in sodium azide to produce an azido compound. The final product, which is AG, is the main material that will be used as a stabilizer in the NRL colloid. Some of the chemical properties were characterized to observe their properties and how they affect the colloid stability of NRL.

The azide group induces a dipole moment by partial displacement of the nitrogen (N) atom, which is the most electronegative in the AG molecule. The AG produced from the azidation process is neutral in pH due to the balance of nitrogen atoms charges (+ve and –ve charges), but it is a polar solvent. 

The presence of a negative (−ve) charge on one of the N atoms in the AG is highly attractive to the positive (+ve) charge in the stern layer of a latex particle, thus forming a dipole moment interaction. This dipole moment interaction is crucial because it ensures that the AG molecules form a layer around the latex particles. This layer increases the colloidal stability of the latex. During freezing, the whole latex will be frozen because the water in the latex serum is frozen.

On the other hand, the water entrapped by the AG around the latex particles will not be frozen due to the anti-freezing properties of glycerol. However, during thawing processes, the frozen latex serum will thaw and the latex particles will be released into the latex without undergoing coalescence. Thus, the frozen latex is said to be regenerated again on thawing. The AG functions not only as a stabilizer but also as an anti-freeze agent. For another explanation, the mechanism of the stabilization of latex particles by an ionic stabilizer during freezing has been explained by Cockbain and co-workers, 1969 [7]. 

#### 3.1.1. FTIR

**Figure 4:**
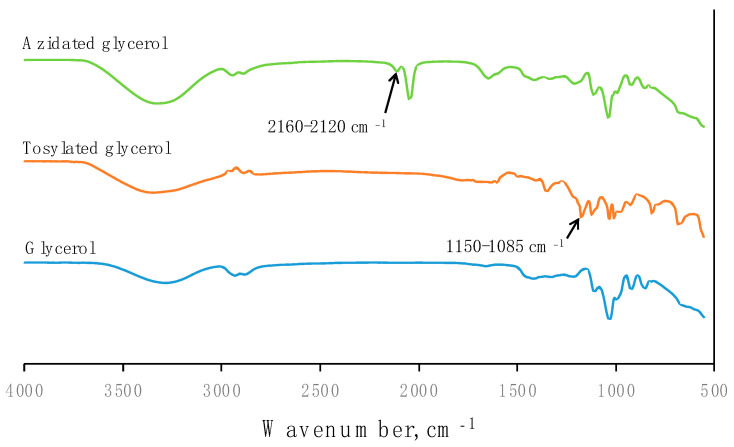
FTIR spectra of glycerol, tosylated glycerol, and azidated glycerol.

The presence of different functional groups of glycerol, tosylated glycerol, and azidated glycerol were analysed by FTIR and their transmittances were compared in Figure 4. In general, a broad peak at the wavenumber range 3000–3700 cm^−1^ detected the presence of the hydroxyl group for all materials. The TSC of AG produced in this study was 80%, so the transmission intensity of the AG spectrum around 3500 cm^−1^ was higher than glycerol due to the other 20% of the aqueous medium containing an OH group. After modification with tosyl chloride, the absorbance peak in the range of 1150–1085 cm^−1^ is related to C–O, as the C–OH of hydroxyl stretching in glycerol has been replaced with C–O stretching of the aliphatic ether in tosylated glycerol. After the modification of tosylated glycerol with sodium azide, the appearance of a peak in the range of 2160–2120 cm^−1^ corresponded to the N=N=N stretching, thus confirming the introduction of an azide group into the AG molecule. 

#### 3.1.2. Morphology of AG

**Figure 5:**
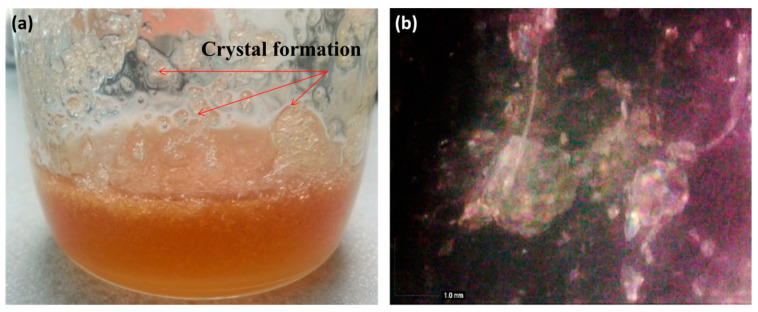
Images of crystal formation of 80% TSC AG by (**a**) the naked eye and (**b**) optical microscopy of aggregation of AG by Dino-Lite at a magnification of 50×.

The morphological behaviour of semi-crystalline structures of AG is shown in Figure 5. AG is a highly water-soluble suspension when mixed with water. However, at 80% (highly concentration) TSC, it will form aggregations that result from the high density of OH groups in AG molecule; therefore, it has a high tendency to form strong associations with itself by hydrogen bonding forces, as shown in the optical microscope image in Figure 5b. 

### 3.2. Effect of Variable Loading of AG on the Colloidal Properties f NRL before and after the Freeze–Thaw Processes

**Figure 6:**
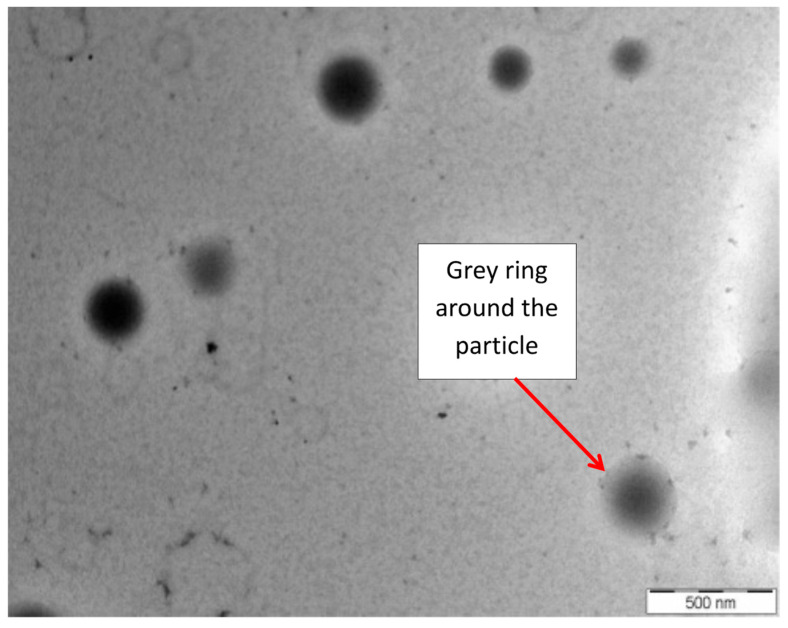
TEM images of NRL particle without stabilizers (0 phr).

Figure 6 shows a TEM image of the NRL particles at 0 phr of AG (NRL without stabilizer). It is observed that grey rings, which are the membrane layers derived from protein lipid, surround the particles. The rubber particles are believed to be covered by some proteins and phospholipids, concerning the colloidal stability of natural rubber latex [17]. Phospholipids are strongly adsorbed to the surfaces of the rubber particles and are believed to be the intermediate by which the proteins are anchored to the rubber particles [18].

**Figure 7:**
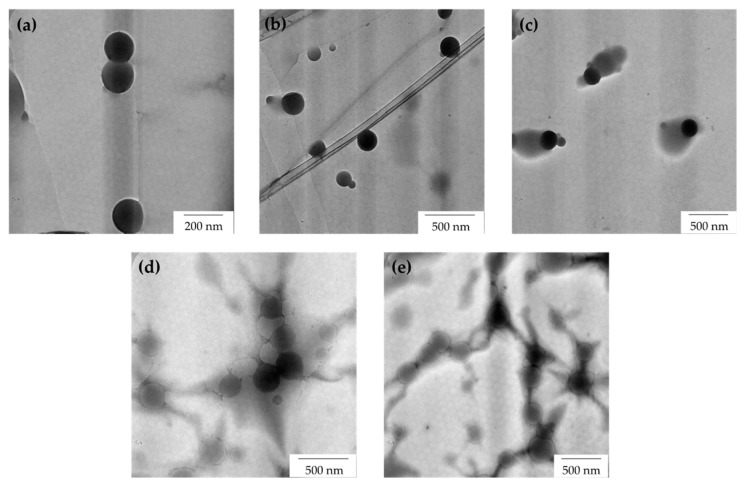
TEM images of AG-stabilized NRL with variable loadings of (**a**) 0.1 phr, (**b**) 0.2 phr, (**c**) 0.3 phr, (**d**) 0.4 phr, and (**e**) 0.5 phr before freezing.

The TEM images of AG-stabilized NRL particles before freezing with variable loadings of 0.1 phr, 0.2 phr, 0.3 phr, 0.4 phr, and 0.5 phr are shown in Figure 7. The particles can be differentiated with two different colour tones which are darker grey and light grey. The darker particles are the latex rubber particles due to the staining process before testing. Osmium tetroxide stains the double bonds of the rubber molecules; hence, all of the rubber particles appear black/darker. Since the latex particles were all stained under the same conditions, the difference in morphology as revealed by micrographs can be attributed to the presence of latex rubber particles with a small percentage of AG at lower loading. Meanwhile, the presence of AG molecules (light grey region) was observed at higher AG loadings. This finding provides fresh evidence of the presence of adsorption of AG molecules around the latex particles. The obvious presence of AG molecules was observed starting at 0.2 phr of AG loading. It showed that the AG molecules started to encapsulate the latex particles even at low stabilizer loading. On the other hand, we also observed the formation of interparticle bridging of AG molecules in between the rubber latex particles at 0.4 phr and 0.5 phr of AG loading, as shown in Figure 7d,e.

A clear interparticle bridging of AG molecules can be seen at higher AG loadings (0.5 phr), as shown in Figure 7e. The latex rubber particles are obstructed from being close to each other by the interparticle bridging of AG molecules. Interparticle bridging has also been observed in colloid systems with high stabilizer concentrations [19]. It is noted that the AG structures were aggregated together to form interparticle bridging, thus acting similar to a barrier obstructing the close approach of latex rubber particles. 

Interparticle bridging by adsorbed molecules gives rise to a strong association between particles. For interparticle bridging to occur, a single molecule must become simultaneously adsorbed on the surfaces of two neighbouring particles. This may occur if the molecule is of sufficiently high molecular mass to bridge the gap between particles, as well as to become firmly adsorbed at the particle surfaces. However, it is also possible that interparticle bridging will occur by association in the interparticle region between separate molecules, some of which are adsorbed on one particle and some on another. In any event, molecules must become adsorbed on the particle surfaces. This implies that the particle surfaces have vacant adsorption sites, or that the adsorption tendency of the molecule is sufficient to displace some of the molecules already adsorbed at the surface. The immediate consequence of interparticle bridging by an adsorbed molecule is effectively that each particle is immobilized relative to its neighbour, and thus it imparts a structure to the latex [3].

**Figure 8:**
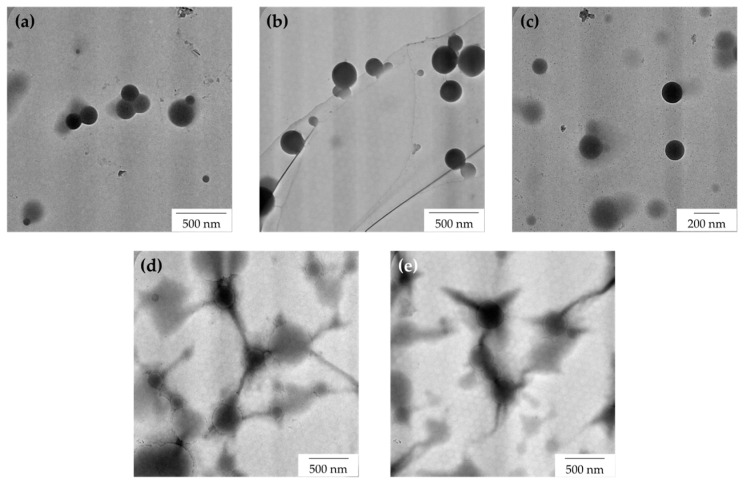
TEM images of AG-stabilized NRL with variable loadings of (**a**) 0.1 phr, (**b**) 0.2 phr, (**c**) 0.3 phr, (**d**) 0.4 phr, and (**e**) 0.5 phr after the freeze–thaw processes.

Figure 8 shows the TEM images of latex particles containing variable loadings of AG after the freeze–thaw processes. From the observations, the morphological behaviour of the regenerated latex containing AG molecules was not affected much by freezing (compared with TEM images of NRL before freezing in Figure 7). This observation suggests that the freezing condition does not much affect the morphological behaviour of NRL containing AG as a stabilizer system. AG molecules are able to protect the latex colloid during freezing and after the freeze–thaw processes.

**Figure 9:**
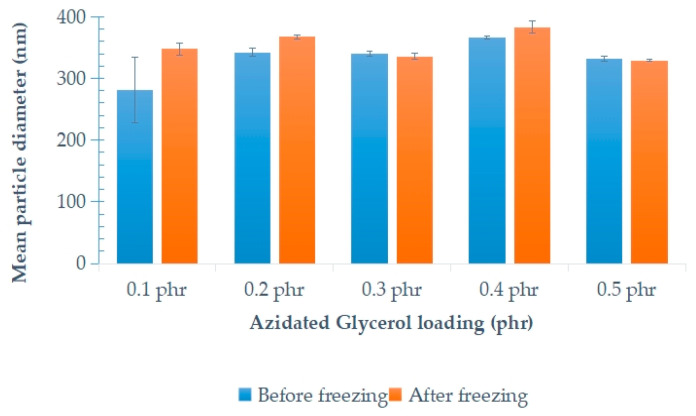
Mean diameter of NRL with variable loadings of AG, before freezing and after the freeze–thaw processes.

Figure 9 shows the mean diameters of the stabilized latex particles before freezing and after the freeze–thaw processes with variable loadings of AG. In general, the mean diameters of stabilized latex particles after freeze–thaw processes were higher compared to the stabilized latex particles before freezing. Under controlled conditions, some of the latex particles coalesced during freezing, and after the thawing process, these coalesced particles did not separate, thus increasing the mean diameter of the latex particles. 

Table 2 shows the zeta potential values of NRL containing AG as a stabilizer system before freezing and after the freeze–thaw processes (regenerated NRL). In general, the zeta potential value is used to predict the stability of colloids such as NRL when stabilizers are added. The zeta potential of NRL is always negative due to the negative charge of proteins and carboxylic groups surrounding the rubber particles. NRL without stabilizers (0 phr) has a zeta potential value of −1.88 mv; an increase or decrease in this value indicates coalescence or repulsion, respectively, among the rubber particles. Nanoparticles with a zeta potential between −10 and +10 mV are considered approximately neutral. If there was no stabilizer present in the NRL (0 phr), this indicates that NRL with zeta potential of −1.88 mV was not yet deprotonated [20]. 

**Figure 10:**
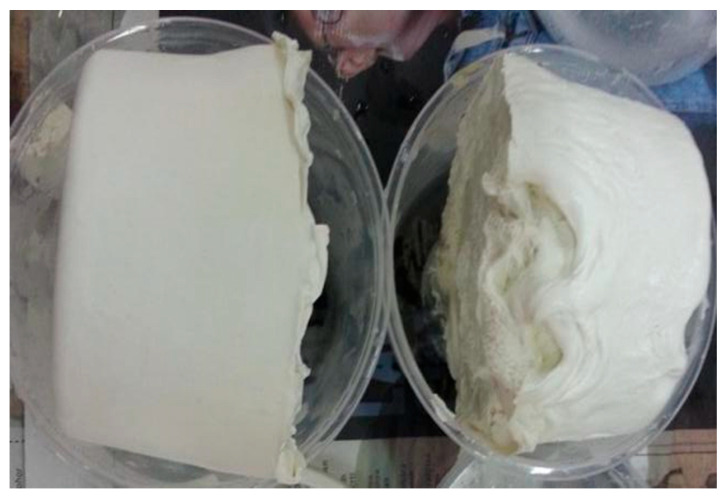
Formation of big lumps of NRL after the freeze–thaw processes at 0 phr AG loading.

After freeze–thawing, the neutral charge of NRL without a stabilizer (0 phr) (zeta potential value between −10 mV and +10 mV, in this case the zeta potential is −1.88 mV) may indicate the inability to maintain latex stabilization after the freeze–thaw cycle, as shown by the presence of a big lump in Figure 10. At 0.1 phr of AG, the latex that was anionically stabilized before freezing has become neutral after the freeze–thaw cycle (zeta potential value between −10 mV and +10 mV). As the AG was increased to 0.2 phr, more AG can stabilize the particles anionically, albeit there is still a reduction in the zeta potential after the freeze–thaw cycle (from −59.11 mV to −56.27 mV). Ammonia presence however was not enough to deprotonate the AG at high phrs of AG (0.3 and above). For AG at 0.4 and 0.5 phr, stabilization, however, was provided by the possible bridges formed between particles by aggregating AG, as shown by the TEM images in Figure 7 and Figure 8. 

A loading of 0.2 phr AG gave the best results before freezing and after the freeze–thaw processes, as it had zeta potentials of −59.63 mV and −56.27 mV before freezing and after freeze–thawing, respectively (nanoparticles with zeta potentials of greater than +30 mV or less than −30 mV are considered strongly cationic or strongly anionic, respectively).

As a control, the NRL without any stabilizer was frozen and thawed. After thawing, it was observed that a big lump of rubber was formed, as shown in Figure 10. The formation of the rubber lump is due to the coalescence of NRL particles after the freeze–thaw process. During freezing, the aqueous medium of NRL is frozen and ice crystals form. NRL particles are trapped in these crystals and are forced to come closer together, thus disrupting the protein cloud surrounding the NRL particles. This broken protein cloud makes the NRL particles more prone to coalesce and form a big lump of rubber after thawing.

**Figure 11:**
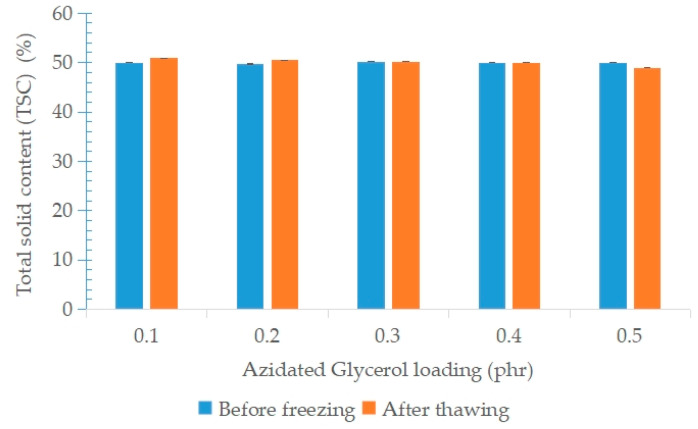
Total solid content of NRL containing variable loadings of AG before freezing and after the freeze–thaw processes.

Figure 11 shows the colloidal properties of NRL containing variable loadings of AG before freezing and after the freeze–thaw processes. In general, addition of AG into NRL was not significant in influencing the TSC of the regenerated latex (freeze–thaw latex). The same trends also are present in the TSC after the freeze–thaw processes. 

**Figure 12:**
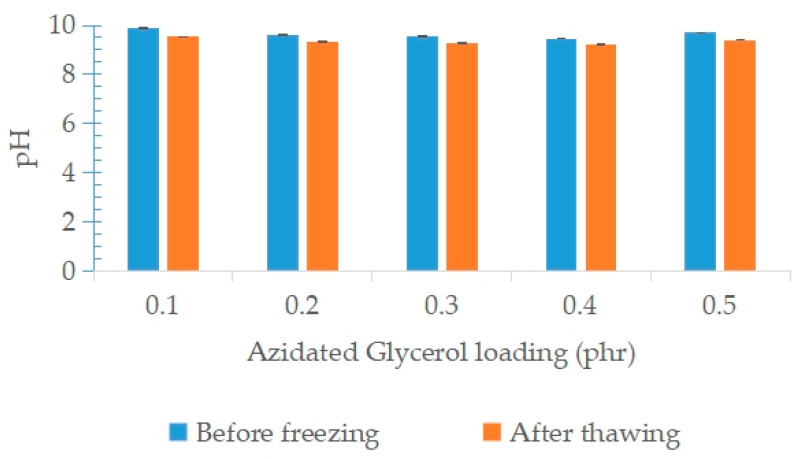
pH of NRL containing variable loadings of AG before freezing and after the freeze–thaw processes.

Figure 12 shows the effect of variable loadings of AG on the pH of NRL before freezing and after the freeze–thaw processes. The pH value of pure glycerol is 5.08 (acidic), meanwhile for AG (80% TSC AG), it is 7.15. On the other hand, the pH value of the 50% diluted NRL before adding any stabilizer was 9.85. The results shown in Figure 12 indicate that the addition of AG did not affect the pH values of the colloid, even at higher loadings. The pH values were slightly reduced after the freeze–thaw processes, which may due to disruption of the protein cloud that influences the negative charge of latex particle.

**Figure 13:**
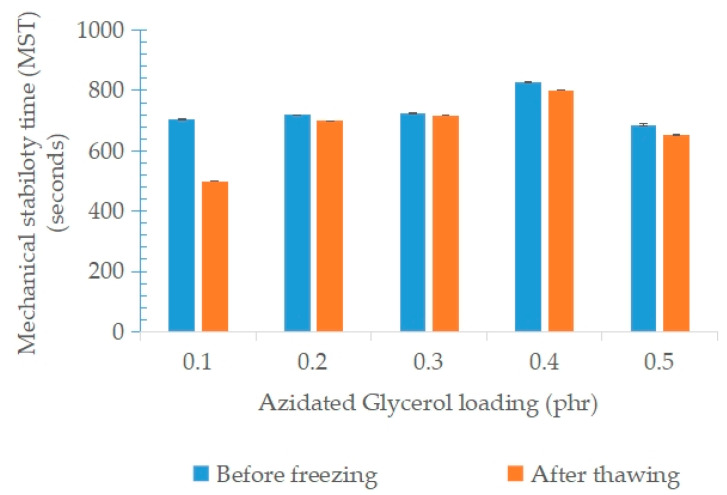
Mechanical stability time (MST) of NRL containing variable loadings of AG before freezing and after the freeze–thaw processes.

Figure 13 shows the mechanical stability time (MST) of NRL containing variable loadings of AG before freezing and after the freeze–thaw processes. In general, increasing AG loading up to 0.5 phr slightly increased the MST from 699 s to 828 s for 0.1 phr to 0.4 phr, respectively. This may be due to the interaction of a negative charge of the N_3_ of the azide group in the AG molecule being more attracted to the positive charge of the stern layer and not disrupting the proteins around the latex particles, thus protecting the rubber latex from faster flocculation. The rates of change in the MST of the regenerated NRL containing AG for each loading was low, especially from 0.2 phr to 0.5 phr of AG. These results show that AG was able to maintain colloid stability after the freeze–thaw process by a low rate of changes in the MST. The formation of a thicker water-bound layer surrounding the latex particle after the addition of AG was able to maintain the colloid stability. This result is explained by the interaction of AG with the nitrogen atoms of proteins surrounding the latex particles. The presence of a thicker/higher water-bound layer able to protect the latex particles at lower temperatures thus maintains the stability of the regenerated NRL colloid at the higher speed of shear. 

The viscosity of NRL containing variable loadings of AG as a stabilizer is lower compared to NRL without AG (0 phr). According to Blackley (1997) [3], the addition of a stabilizer was to slow down the increases in the viscosity of the NRL while mechanical agitation continued. Additionally, the slight increase in latex viscosity while increasing AG loading provides evidence of the effects in the interfacial region between the particle and the aqueous phase. The interparticle bridging by AG molecules at higher loadings can increase the viscosity of the latex due to the dispersion medium being entrapped in the particle agglomerates, thereby increasing the volume fraction of the dispersed phase in the latex [3]. 

**Figure 14:**
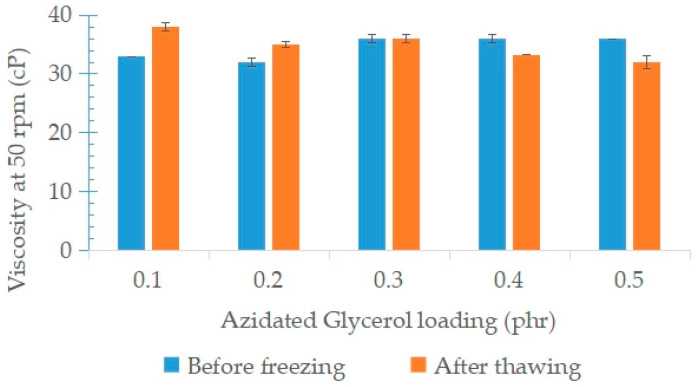
Viscosity of NRL containing variable loadings of AG before freezing and the after freeze–thaw processes.

The viscosity of NRL colloids containing 0.4 phr and 0.5 phr of AG after freeze–thawing reduced compared to the NRL before freezing, as shown in Figure 14. This behaviour shows that at higher AG loadings, the particles of regenerated NRL are free to move in the colloid without any restrictions. 

## 4. Conclusions

The addition of AG as a stabilizer system has been shown to improve the colloid stability of the NRL after the freeze–thawing process when compared to NRL without the addition of a stabilizer (which formed a big lump after the freeze–thaw process). Frozen NRL (ice cream-like texture) was successfully produced and could be thawed and re-frozen again for the subsequent manufacturing processes. The introduction of an azide group into the AG molecule was confirmed in the FTIR spectra by the appearance of a peak in the range of 2160–2120 cm^−1^, corresponding to N=N=N stretching. Modifying NRL with AG did not significantly influence the TSC of latex. Increasing the AG up to 0.4 phr waspo shown to increase the MST. NRL with AG at 0.2 phr resulted in the highest anionically stabilized latex, as indicated by the zeta potential value of −59.63 mV (before freezing) and −56.27 mV (after thawing). With all the properties observed, it is expected that frozen NRL stabilized by AG is suitable to be transported by sea.

## Figures and Tables

**Table 1:** Formulations of NRL with variable loadings of AG.

Ingredients	Parts per Hundred Rubber (phr)
50% NRL	100
AG	0
	0.1
	0.2
	0.3
	0.4
	0.5

**Table 2:** Zeta potential values of AG-stabilized NRL particles before freezing and after the freeze–thaw process.

Stabilizer Loading (phr)	Zeta Potential (mV)	
	Before Freezing	After Freeze–Thaw
0	−1.88	Big Lump
0.1	−15.11	−6.94
0.2	−59.63	−56.27
0.3	−2.50	−5.30
0.4	−2.67	−14.93
0.5	−1.66	−1.86

## Data Availability

The data that support the findings of this study are available on request from the corresponding author and Siti Nuraya Abd Samat.
